# Supplementary material for: Localization of infection in neonatal rhesus macaques after oral viral challenge
Source: PLoS Pathog. 2021 Nov 18;17(11):e1009855. doi: 10.1371/journal.ppat.1009855 (PMC8639050; doi:10.1371/journal.ppat.1009855)
Supplement: S1 Table — *Day of harvest, date of harvest. (PDF) [file ppat.1009855.s001.pdf]

**Animal**	**Experiment**	**Sex**	**Age at challenge (days)**	**Age at necropsy (days)**	**Teeth erupted**	**Virus used /(Viral stock of SHIV-1157ipd3N4 used)***	**$TCID_{50}$ Cumulative dose of SHIV (x 10^6 $TCID_{50}$ - All feeds)**	**Viral load (copies/mL)**
A19X038 (4 hours)	PET/CT	M	8	8	No	PA-GFP-BaL	NA	NA
A19X042 (2 hours)		M	1	1	No	PA-GFP-BaL	NA	NA
A19X073 (2 hours)		M	2	2	No	PA-GFP-BaL	NA	NA
A19X074 (4 hours)		M	2	2	No	PA-GFP-BaL	NA	NA
1	LICh pilot	F	5	9	No	LICh	NA	NA
13	53 hr low dose dual challenge	F	10	12	No	LICh + SHIV-1157ipd3N4 (Day 7 ǀ 7/30/2012)	31	2212
10	96 hr high dose dual challenge	F	7	11	No	LICh + SHIV-1157ipd3N4 (Day 7 ǀ 7/30/2012)	31	213341
17		M	11	15	No	LICh + SHIV-1157ipd3N4 (Day 7 ǀ 7/30/2012)	31	254850
22		M	7	11	No	LICh + SHIV-1157ipd3N4 (Day 5 ǀ 9/3/2016, Day 8 ǀ 9/6/2016	32	1408
23		F	8	12	No	LICh + SHIV-1157ipd3N4 (Day 8 ǀ 11/22/2016, Day 9 ǀ 11/23/2016, Day11ǀ11/25/2016, Day 12ǀ11/27/2016	34	1665
25		M	5	9	Yes	LICh + SHIV-1157ipd3N4 (Day 13 ǀ 4/3/2017)	36	10731
26		M	4	8	Yes	LICh + SHIV-1157ipd3N4 (Day 13 ǀ 4/3/2017)	36	21223
27		M	6	10	No	LICh + SHIV-1157ipd3N4 (Day 9 ǀ 3/30/2017, Day 13 ǀ 4/3/2017)	36	1245
28		F	3	7	No	LICh + SHIV-1157ipd3N4 (Day 17 ǀ 4/7/2017, Day 13 ǀ 4/3/2017)	36	17003

*Day of harvest, date of harvest for SHIV-1157ipd3N4
