# Supplementary figures and images for: Localization of infection in neonatal rhesus macaques after oral viral challenge
Source: PLoS Pathog. 2021 Nov 18;17(11):e1009855. doi: 10.1371/journal.ppat.1009855 (PMC8639050; doi:10.1371/journal.ppat.1009855)

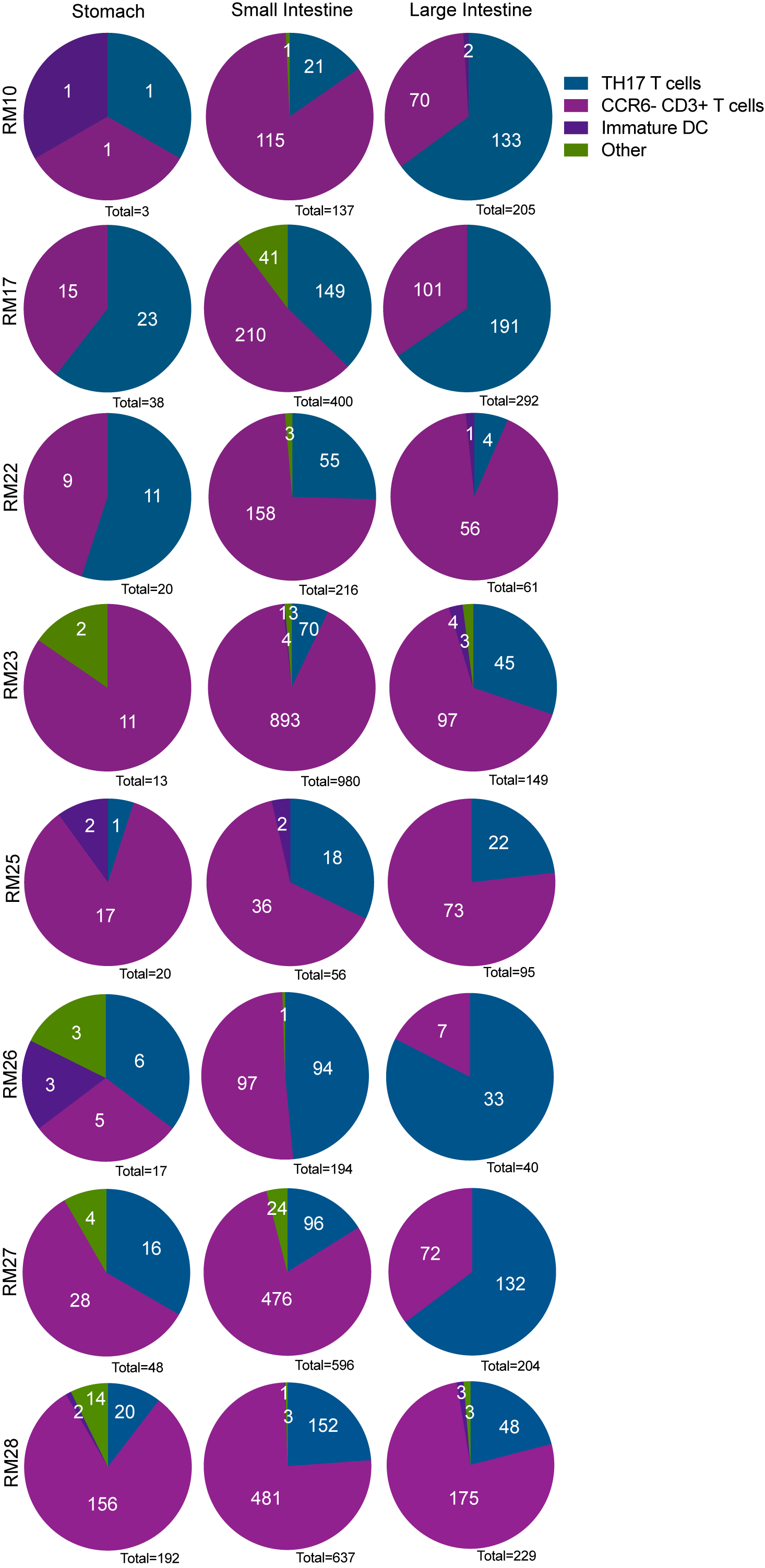

Supplement: S1 Fig — Quantification of SHIV-1157ipd3N4 infected cells found in the GI tract of each animal examined in Figs 3 and 4. Graphs depict the percentage of infected cell types as parts of a whole in each individual animal identified by fluorescent microscopy. Infected cell types were categorized as five cell types: T cells (CD3+), TH17 T cells (CD3+, CCR6+), CCR6- CD3+ T cells, Immature DCs (CD3-, CCR6+), Other (CD3-, CCR6-). Total cell counts were taken in five 40x panels in every animal. (TIF) [file ppat.1009855.s002.tif]
