# Supplementary material for: Localization of infection in neonatal rhesus macaques after oral viral challenge
Source: PLoS Pathog. 2021 Nov 18;17(11):e1009855. doi: 10.1371/journal.ppat.1009855 (PMC8639050; doi:10.1371/journal.ppat.1009855)
Supplement: Attachment — Submitted filename: Response to reviewers comments.pdf [file ppat.1009855.s003.pdf]

Northwestern Medicine
Feinberg School of Medicine

Thomas J. Hope,
PhDProfessor
Principal Investigator
Cell and Developmental Biology
Northwestern University Feinberg
School of Medicine

312.503.2337 office
312.908.2528 fax
312.503.1360 direct
thope@northwestern.edu

Simpson Querrey, 6th floor
303 East Superior Street
Chicago, Illinois 60611

Northwestern

Dear Editor,

We are pleased to resubmit this article entitled "Localization of infection in neonatal rhesus macaques after oral viral challenge" (PPATHOGENS-D-21-01555 - [EMID:3e48961f099a383b]). We would like to thank the reviewers for their thoughtful comments to improve the manuscript.

We believe that the revised manuscript and figures address the concerns and comments of the reviewers. Wehave changed the language of, edited, and added sections of the introduction, results, discussion, and methods for clarity. Specifically, we have removed the previous Figure 3 depicting microscopy data for mCherry and Luciferase staining to examine LICh transduction in tissues. In addition, the new Figures 3 and 4, SFig1, and Table 3 have been revised to indicate that "Other T cells" are CCR6- CD3+T cells; likewise, Tables 2 and 3 have been edited to depict exact number of cells found in each location to bring clarity and indicate the magnitude of infected cells. Supplemental table 1 has been edited to include every animal used in this study. Our methods and discussion have been revised to bring clarity to our experimental design and discuss limitations of our specific model of bottle-fed oral viral challenge. We hope that the manuscript is significantly improved and is acceptable for publication in Plos Pathogens.

Responses to specific reviewer comments are presented in italics below.

Part I - Summary
Please use this section to discuss strengths/weaknesses of study, novelty/significance, general execution andscholarship.

Reviewer #1: his manuscript reveals novel information on virion trafficking and initial infected cell types in the infant gut after oral virus challenge in bottlefed infant monkeys. This is important work that may aid in developing interventions for this most common route of HIV infection in infants that is currently not addressed by antiretroviral-based prevention alone. Yet, there are limitations in the model that should be further discussed and indicated as limitations, such as the impact of breast milk itself on the virion trafficking and infection of cells in the mucosa.

We appreciate the supportive words relating to our "important work that may aid in developing interventions" for this route of HIV infection in infants. We believe we have addressed the limitations in the model, specifically regarding the impact of breast milk on virion trafficking and infection of cells, and we thank you for this feedback.

Reviewer #2: HIV-1 infection by breastfeeding remains a major cause of new pediatric infections. Defining the kinetics of virus dissemination and identifying early sites of virus replication may inform novel prevention strategies. The study by Taylor et al. utilized the neonatal rhesus macaque model to address

these questions. Applying PET/CT and fluorescent microscopy, the investigators can demonstrate that virus rapidly disseminate from the oral cavity to the rectum within 4 hours of virus feeding. By 96 hours, virus can be detected throughout the entire gastrointestinal tract, with the majority of infected cells representing non-Th17 T cells in the small and large intestine.

The strength of the study lies in capturing of very early (4 hours) viral foci at sites distal form the virus exposure site by applying PET/CT in combination with fluorescent deconvolution microscopy. The in vivo analysis of virus dissemination is not an easy endeavor. The thoroughness of the evaluation of tissue sections from the entire GI tract to identify the phenotype of virally infected cells is acknowledged. However, the finding that T cells represent the main infected cell type at 96 hours is not necessarily novel (Wang at al, 2010, Blood, 116:4168; Amedee et al., 2018, AIDS Res Hu Retroviruses, 34:286), even in the light of different routes of virus exposure. The fact that Th17 CD4+ T cells did not represent the majority of infected T cells is interesting and should be discussed in the context of animal rearing. Ardeshir et al. demonstrated previously that the establishment of Th17 cells is more pronounced in breastfed vs. nursery-reared infant rhesus macaques (Ardeshir et al., 2014, Sci Transl. Med., 6:252ra120).

Thank you for the supportive words regarding our technical approach to study viral distribution after oral viral challenge. The novel findings of our paper are that virions distribute and penetrate into the rectal mucosa four hours after oral viral challenge and that the largest foci of infection at 96 hours are in the gut, specifically the small intestine. We acknowledge that finding that T cells are the largest phenotype infected at 96 hours is not necessarily novel and give reference to other papers that have found similar results, including the Wang et.al 2010 and Amedee et.al. 2018. We also find that Th17 T cells not being the majority of infected T cells is interesting, especially since we have previously published that in adult macaques Th17 are the majority of infected cells in the female reproductive tract during vaginal SIV infection. We have addressed your concerns regarding this finding in the manuscript.

Reviewer #3: This manuscript by Taylor et al utilizes recent technological advancements to address the question of how the SIV/SHIV virus infects an infant macaque monkey following oral exposure in the form of formula feeding. There are a number of findings in the paper that validate previous publications, as well as to provide additional insights into what is occurring at times following oral exposure of virus or viral reporters to infant macaques. Overall, while the technology utilized does provide interesting insights, there are a number of major issues that dampen the overall enthusiasm.

Part II – Major Issues: Key Experiments Required for Acceptance
Please use this section to detail the key new experiments or modifications of existing experiments that shouldbe <u>absolutely</u> required to validate study conclusions.

Generally, there should be no more than 3 such required experiments or major modifications for a "Major Revision" recommendation. If more than 3 experiments are necessary to validate the study conclusions, thenyou are encouraged to recommend "Reject".

Reviewer #1: 1. while these studies are very revealing in the trafficking of virions and the initial infected cells, it may not represent the low dose exposure of breast milk feeding, as only 10% of breastfeeding infants of HIV+, untreated moms will become infected over the entire period of breastfeeding. The differences of this model and physiologic breastfeeding should be discussed, in particular, why so few infants become infected over the period of breastfeeding even in the absence of antivirals yet this model indicated that all challenged infants had virus-infected CD4+ T cells in the GI mucosa.

Thank you for this statement. We agree that the difference between our model and physiologic breastfeeding should be discussed. We have added a section to the discussion addressing this, which can be found in lines 537-555:

"It is important to note that in our model of oral viral challenge, the viral inoculum was diluted for delivery in Pedialyte (see Methods), which is not physiologically representative of breast milk. Breast milk components have been shown to serve as antimicrobial and anti-inflammatory agents [51]. Lactoferrin has antiviral properties that can inhibit HIV-induced cytopathic effect [52,53]. Oligosaccharides found in breast milk have been shown to be effective inhibitors of HIV. Breast milk also contains an abundance of maternal antibodies that could protect infants against infection [51]. These protective components most likely are the reason not every breastfed baby acquires HIV when orally exposed. The lack of these protective components in our model could partially explain why we found infected cells in the GI mucosa in every animal in our study."

2. The statistics need to be reviewed and further described, as I am not sure how an FDR-corrected significant p value can be achieved with only 4 animals using paired tests (since the observations come from the same animal). The multiple images reviewed should be averaged for each infant as opposed to used as multiple data points.

We agree with the reviewer that the low number of animals poses a challenge when analyzing the results, which is why we have utilized the analytical approaches best suited for the evaluation of this unique data set. Unfortunately, we did not describe our approach well enough to be clear and justified to the reader. In the revised manuscript, we have updated the methods and description to inform the reader about the complexities and rational for the analysis. However, we respectfully disagree with the reviewer about changing or modifying the statistical methods used. Specifically, the focus of these analyses was to explore statistically significant differences in the anatomical distribution of virions and their characteristics, not the differences between animals. With this objective, due to our low number of animals, we performed an exhaustive imaging of tissues to achieve an extensive sampling of virions in each tissue of the animals analyzed.

In the different analyses we used different mixed-effect models, as described in the methods, always using each animal as random effect to control for the lack of independence of the data sampled within each animal. Therefore, the results reflect and account for this fact. Afterwards, within the different models used and depending on the nature of the data analyzed, we performed all possible contrasts between every location within such fitted models to analyze statistically significant differences in the number of virions and/or their characteristics in each location. In order to do so, we used the Benjamini-Hochberg (BH) Procedure, as described in the methods, which controls for False Discovery Rate (FDR). On the contrary to what the reviewer questions about significant p-values for only 4 animals, when using BH, there is no reason why the sample size is relevant in expecting or not significant differences, as it is just a method to avoid Type I errors. Moreover, the power of our analysis is derived from the extensive sampling of virions in different tissues through multiple images from the animals, calculating averages per animals would defeat that purpose, gives us a very significant result without proper testing, and wouldn't allow us to test for our main interest, the differential anatomical distribution of the virions after mucosal exposure.

3. Tables 2 and 3 should indicate magnitude of infected cells identified in each tissue, even though it seems the technologies are quantifiable. And perhaps as a heat map instead of a table would be a better visual.

Thank you for this suggestion. We have changed both Tables 2 and 3 to depict the number of infected cells counted in each tissue type. While a heat map would be a good representation of the data, we decided to keep the tables in order to show exact numbers of cells found.

4. As the LICh experiment had many technical challenges presented in the results and few reliable data

points, the data from this approach should be removed, and this employed approach can mainly be included in the methods and discussion

Thank you for this suggestion. We have removed figure 3 as per the suggestions of the reviewers. We have minimized the results and discussion surrounding LICh to a few sentences in the results, maintained LICh data in Tables 1 and 2 for data representation, and have a short section surrounding LICh in the discussion.

5. The method of bottle-fed inoculation includes pedialyte as the diluent, which is not a physiologic representation of breast milk or even formula. The authors should discuss the limitations of this approach and how the virion distribution and infections may have changed with the virus diluted in breast milk, which has a number of innate anti-microbial proteins, lipids, as well as maternal antibodies.

The Pedialyte, while not formula or breast milk, was used only for the viral challenges themselves as a fluid diluent for the challenge inoculum. The animals used in the LICh and SHIV-1157ipd3N4 experiments were provided formula for every feeding that was not a part of the viral challenge. We have previously shown that the use of PET/CT allows for an unbiased tracking of viral distribution throughout the gastrointestinal tract by locating areas of radioactivity (Taylor et.al., 2021). From these studies, we garnered that individual viral particles follow the path of the gastrointestinal tract and the flow of the diluent used; in Taylor et.al., 2021, PBS was used as the diluent. While the use of Pedialyte as a diluent may have limited effect on the distribution of the virus, the point made about the difference in infections due to the lack of antimicrobial components found in breast milk is an important one to address. We have added this to the discussion in lines 537-553: "It is important to note that in our model of oral viral challenge, the viral inoculum was diluted for delivery in Pedialyte (see Methods), which is not physiologically representative of breast milk. Breast milk components have been shown to serve as antimicrobial and anti-inflammatory agents [51]. Lactoferrin has antiviral properties that can inhibit HIV-induced cytopathic effect [52,53]. Oligosaccharides found in breast milk have been shown to be effective inhibitors of HIV. Breast milk also contains an abundance of maternal antibodies that could protect infants against infection [51]. These protective components most likely are the reason not every breastfed baby acquires HIV when orally exposed."

Reviewer #2: 1. Although the authors state that mesenteric lymph nodes were only collected from two animals, what was the rationale of not including lymph nodes in this analysis?

Thank you for this comment. The mesenteric lymph nodes from the other animals were unfortunately not collected at the time of necropsy. For clarity, mesenteric lymph node data has been removed from the manuscript text and tables.

2. Tissue sections were stained with CD3 and CCR6 to distinguish between T cells, Th17 cells and immature DC. The addition of a CD4 antibody would have allowed to quantitate the number of CCR6+CD4+ T cells (likely Th17+) of total CD4+ T cells which might more accurately reflect CD4+ Th17 cells as CCR6 is also expressed by $\gamma\delta$ T cells and certain CD8+ T cells. Along the same lines, the statement that the results of the study provide insight into mechanisms (line 441) of acute infection after oral SHIV exposure is not justified and would warrant a more detailed analysis of various CD4 populations and their viral coreceptors

Thank you for this comment. It has been previously reported that HIV and SHIV need to bind both CD4 and CCR5 or CXCR4 in order to infect a cell. Furthermore, it has also been shown that CD4 is not only downregulated, but also degraded during HIV, SIV, and SHIV infection. Because of this, staining for CD4 in tissue sections of infected animals is not feasible. CD8 has not been shown to be a coreceptor for HIV,

SIV, or SHIV, therefore the AG3+ cells that we observe cannot be CD8+ T cells. γδ T cells, while a minor subset of T cells, may be infected in this model. We have discussed this possibility in the discussion (lines 575 – 578):

"Likewise, the CD3+ CCR6+ AG3+ cells that we observed throughout the oral mucosa and GI tracts of the neonatal RMs could be γδ T cells as they also express CCR6. While γδ T cells are a minor subset of T cells in both humans and RMs, they have been shown to be infected by HIV and SIV [55,56]."

3. The timing of feeding in the eight animals that were infected with SHIV1157ipd3N4 needs to be clarified. The Method section lists that infants were few eight times with 5 ml of the virus. It is important to know whether the feeding interval. If the feeding was spread out over multiple times, it is feasible that the 96-hour time point could refer to a few hours post the last virus exposure up to 96 hours depending on what exposure resulted in infection.

Thank you for this comment. We understand that how the feeding was performed in the original manuscript was not clear and led to confusion. There were multiple feedings that occurred for the 96 hour experiments. We have clarified this in the methods section, which can be found from lines 658-662:

"The remaining eight animals were challenged via eight feedings of 5mL SHIV-1157ipd3N4 over a span of two days (for a total of 40mL of SHIV115ipd3N4 over the course of the experiment) + one dose of 8mL LICh, which was included in the final feeding (Supplemental Table 1). Animals were sacrificed 96 hours after the initial viral challenge feeding."

4. Please add a statement to the rearing of the neonatal macaques, presumably nursery-reared? In addition, please also list the age of the 4 animals used for PET/CT studies and for the two animals in the LICh only studies.

Thank you for this comment. We have discussed nursery-rearing of the neonates in two sections: Discussion, the effects of nursery-reading regarding Th17 T cells (lines 566-573: "It has been shown that breastfed and bottle fed RMs develop distinct immune systems and gut microbiota, with a major difference being in the development of Th17 T cells [54]. Ardeshir et.al. demonstrate that breastfed RMs develop robust memory and Th17 T cell populations whereas bottle fed RMs did not [54]. This distinction is very important and could explain the results shown here that using this model of oral viral challenge in nursery-reared RMs, Th17 T cells were not found to be the largely infected." *and in Methods, regarding the physical rearing of the neonates (lines 650-652;* "All 14 animals used in the experiments described in this manuscript were nursery-reared by veterinary staff at either Texas Biomed or NIRC." *We have also included all information for the animals used in this manuscript in the supplemental table 1.*

5. Line 348: CCR5 is certainly a coreceptor for HIV, but CCR5 is not commonly referred to as a mucosal homing marker. Although activated (and CCR5+) T cells are more commonly found at mucosal sites, the integrin alpha4beta7 would be a more appropriate mucosal homing marker for intestinal tissues.

While alpha4beta7 is certainly a mucosal homing marker for the intestinal tissues, CCR5 cells are greatly enriched in the majority of various mucosal sites. Because it was unknown how the virus would distribute throughout the oral mucosa and the GI tract after oral viral challenge, examining alpha4beta7 was not deemed appropriate for this study. Furthermore, once the cell has reached the mucosa, alpha4beta7 can be downregulated. The interaction of alpha4beta7 with MADCAM in post capillary venules facilitates in certain mucosal sites like the gut. CCR5 plays a role in cell proximity to the lumen and inflammatory sites. Future studies examining the gut specifically after oral challenge could certainly examine the role of alpha4beta7, however this is out of the scope of this paper. In regard to calling CCR5 a mucosal homing

receptor, the language has been changed to reflect your comment. These changes can be found on lines: 441-445:

“Importantly, most of the T cells in the tonsil do not express the chemokine receptor CCR5, which is a required co-receptor which in combination with CD4 is required for the virion to functionally fuse with the target cell membrane. CCR5 is enriched at mucosal sites, low levels of CCR5 are observed in lymphatic tissue such as lymph nodes and tonsil.”

Reviewer #3:

1. There is a concern with regard to using radioactive copper as a way to identify entry points of SIV/SHIV in orally inoculated infant macaques. Looking at the images in Figures 1 and 2, it appears that the virus with radioactive copper simply followed the liquid down the digestive tract unless it hit an air bubble (or went to the large intestine). I think this says that the virus can potentially make it down that far in the amount of time assessed, which is informative. However, the authors should be careful not to over interpret the location of the radioactive copper or the GFP as indicative of the most likely entry points since the entire digestive tract appears to be bathed in it.

Thank you for this comment. The virus uses diffusion within fluids to distribute within the available space after exposure. Neonates only consume fluids. Those fluids are primarily adsorbed in the large intestine. Our method allows us to follow the localization of the 64Cu signal. But the key to the rigor of this methods is we can then identify intact viral particles within the tissue identified by radioactive signal. That validation demonstrates that we are observing how viral particles will distribute under the conditions of the anatomical exposure. Before these technologies were utilized, our laboratory searched for PA-GFP-BaL virions for years and did not find any evidence of virions despite those experiments also using a fluid to deliver the virus into the body of RMs. Why, because we were looking in the wrong place.

We agree with reviewer #3 that the amount of fluid can have an impact on fluid (and diffusing virus) distribution. For example studies reveal that with more fluid, there is greater distribution after rectal instillation. This was shown with methylene blue distribution comparing 1 ml and 3 ml after rectal instillation (Smedley et al, 2014, PLOS one). In Taylor et.al. 2021 we recently reported the distribution of HIV utilizing the PET/CT guided necropsy approach used in herein and could evaluate the distribution of viral particles after rectal instillation and showed the extent of distribution could actually be increased by the presence of dIgA specific to the virus. However, in this case we are dealing with neonates which are only consuming fluids. Therefore, the volume relating to the natural consumption of fluids in neonates is less relevant in this case. Our observations reveal that viral particles in orally consumed virions can remain intact and penetrate the gut mucosal barriers. We believe that this observation is of interest to the HIV research community.

Please refer to lines (580-581) “These data demonstrate that HIV virions can distribute throughout and penetrate the entire GI tract while remaining intact at a very rapid rate hours after oral viral exposure. These initial observations reveal where the intact virions can go after an oral exposure.”

2. Figure 2 E, F and G are clearly impactful and potentially important figures in this manuscript. However, there are a few issues that are concerning. First, the data is dependent on photo micrograph from Figure 1C being clear and easy to interpret, however only one GFP positive virus is easily discernable and that is in the inset. Without clearer pictures from different tissues it is difficult to know how to interpret data from graphs. Second, the y axis for Figures 2E and 2F are easy to understand. Although a brief description is present in methods for total virions counted, this is not sufficient for understanding how the different tissues were evaluated. For example, why do the methods say 20 or 22 images, shouldn’t that have been the same number for each tissue if you were counting total numbers of GFP positive spots? The exact area

being evaluated would also be good to know. Third, the differences from 2 to 4 hours resembles what one would expect for a virus particle that was traveling with formula. It is not clear that this says very much about viral entry, I think it says something about potential for viral entry.

Thank you for your comment. We have reviewed the image of the micrograph in Figure 1C, and with the quality of image required for publication, there are more discernable virions in the image. We believe the discrepancy is due to a decrease in quality of microscopy images upon creating the file for reviewers. The number of images were controlled for in our statistical analysis for quantifying the number of virions that were found throughout the oral and gut mucosa. This information can be found in our Statistics section of Methods on lines 771-774.

"Virion counts per necropsy time were modeled using a negative binomial generalized mixed model to test for differences between anatomical locations, controlling for the number of images taken per animal and tissue."

For the area of the tissue that was specifically examined, the technologies of PET/CT allow us to perform an unbiased approach to identify how the virus distributes through and penetrate the mucosa. Because of this, we selected areas based on the intensity of radioactivity observed. Then 20-22 images were taken in an unbiased manner by simply following the linear path of the luminal surface. An important consideration that may not be appreciated by the reviewer is that we are at the mercy of the irregularity of the luminal surface. This information can be found in our previous publication (Taylor et.al. 2021) and in lines 723-728:

"The 20-22 images were taken in an unbiased manner by following the linear path of the luminal surface of the mucosa with the distance between each image dictated by bleaching caused by photoactivation. Images were analyzed with softWoRx software (Applied Precision, Issaquah, Washington). Number of total virions and penetrating virions were counted. Virions were defined by existing in more than three Z planes and the post-activation signal had to be at least three times higher than the pre-activation signal."

For the issue of viral entry, we evaluate the number of viral particles entering the tissue and their depth of penetration. We can say the virions the virions have entered the tissue and have the potential to initiate infection. We will make this distinction clear to the reviewer.

3. Figure 3. The LICh vector analysis was problematic for reasons raised by authors. Furthermore, there does not appear to add anything substantial to conclusions from the manuscript. It seems that all figures associated with this can be removed without impacting any of the conclusions from the manuscript. Use of vector can still be mentioned, but in my opinion a clear explanation of why these data are included is needed if they are to remain.

Thank you for this suggestion. We have removed Figure 3, which shows the data collected from LICh experiments, as per the suggestions of the reviewers. We have minimized the results section on LICh data to a few sentences in the results, maintained LICh data in Table 1 and 3 for data representation, and have a short discussion surrounding LICh in the discussion section.

4. CCR6. Microscopy data for CCR6 is not convincing. Therefore, conclusions regarding infection of the different cell subsets is called into question. Can better micrographs be utilized instead of what is included here or were the positive cells just slightly above the background?

Thank you for this suggestion. Upon review of the images of CCR6 shown, we believe that the publication quality images show convincing CCR6 staining. It is possible that during the review process that the quality of image had decreased. We do not believe it appropriate to choose different microscopy images as

the images shown here are representative images as opposed to images that contained the largest number of CCR6+ AG3+ cells. The data is captured in the quantification of the images, the representative image is to help the reader understand the nature of the images generated.

5. Detection of SHIV infected cells in the intestines after 4 days might reflect more rapid replication here in lymphoid tissues associated with gut mucosa. Therefore, you may not be looking at entry, but rather a viral replication site that is replicating a viral population that entered at a different site. This is because 4 days is quite a long time from when the macaque was infected. Is there any data from earlier time points that could help to sort this out? Also, and this applies to comment 2 above as well, it would be great to have more info in figure legends regarding what it means when the data reports on 'number of cells'.

Whether or not we are examining viral replication at 96 hours in the gut mucosa versus viral entry is a very valid statement. However, we respectfully disagree that 4 days is a long time. If we assume that the viral lifecycle is 24 hours then at the 96 hour time point the virus has had time to complete 4 replication cycles. The possibility that infection was initiated by a viral particle that had reached the gut mucosa after traversing through the alimentary canal. In our discussion, on lines 457-483, we discuss that these studies cannot indicated whether the infected cells located in the intestines at 96 hours are from the challenge inoculum or a result of viral replication:

"While we found the majority of SHIV-1157ipd3N4 cells in the small and large intestine, it is important to note that we cannot determine with our methods if these large foci of infection represent the initial sites of infection at the portal of transmission or a location rich in target cells that can foster high levels of viral replication after initial expansion and replication in the oral cavity. It seems the large number of expanding, substantial infectious foci in the gut most likely initiated in the gut considering the short four-day period between drinking virus containing fluid and necropsy. The efficient movement of the virus containing fluid we documented throughout the alimentary canal four hours after drinking and our ability to identify intact viral particles penetrating the intestinal mucosa likewise support a model where oral exposure of virus containing fluid can utilize the intestine as a portal of transmission.

Another retrovirus known to be milk transmitted is the mouse mammary tumor virus (MMTV) that disseminates through the GI tract and directly infects immune cells in the Peyer's patches of the gut, suggesting that other breast milk-transmitted retroviruses may also have this capability [43,44]. Although current models of MMTV acquisition suggest that this process takes place through cell-associated virus (infected cells in milk) which could withstand the acidic pH of stomach acid better than cell-free virus [45]. However, our observations clearly demonstrate the viral particle can reach the intestinal compartment intact, without being cell associated, agreeing with previous results from Baba et. al. which demonstrated that cell-free SIV is transmitted orally in neonatal rhesus macaques [38]. Providing the challenge virus in formula may have played a role in buffering the mucosal environment slowing virion lysis and degradation. Therefore, our PET/CT and microscopy data, taken together with the knowledge that the gut is a target rich environment for viral infection is consistent with a model where the SHIV-1157ipd3N4 infected cells we found in the intestines originated from virus in the challenge inoculum utilizing the gut as the primary portal of transmission."

We have added more to this discussion to emphasize this inability to differentiate whether the cells found were from the challenge inoculum or viral replication. Please see the following that was added in lines: 483-494:

"However, due to the replicative properties of SHIV-1157ipd3N4 we cannot rule out the possibility that the infected cells found in the small and large intestine originate from virus that had disseminated from the portal of transmission in the oral cavity. But assuming a 24 hour replication cycle for SIV, it seems unlikely that 96 hours is enough time to generate the large foci of infected cell we observe in gut mucosa. It is notable that we observe similar size foci of infection at 96 hours after vaginal or rectal challenge. It is

notable that Amadee et al also detected gut infection by RNA PCR 96 hours after oral challenge, but interpreted the gut signal to be a consequence of virus dissemination from the oral cavity because viremia could also be detectable at the 96 hour timepoint. However, our observation of intact viral particles penetrating the gut mucosal barriers after oral challenges increases the potential that oral exposure can initiate infection in the gut."

We have also updated Tables 2 and 3 by replacing the symbols showing areas containing infected cells to the number of infected cells quantified in all of the images for transparency.

6. Subtype C SHIV. It is not clear why the authors did not simply use a SIV for this study. Transmission data with a SHIV might differ from a virus that naturally infects nonhuman primates. A better explanation with regard to why this virus was utilized is warranted.

*Thank you for this suggestion. For the PET/CT study, we utilized HIV_{BaL} that contained a PA-GFP-Vpr for labeling purposes and for the 96-hour experiments examining viral replication and infected cell phenotypes we used SHIV-1157ipd3N4. We have not been able to generate PA-GFP-SIV particles with PA-GFP levels as high as our HIV_{BaL}. Additionally, these viruses were chosen because both contain an HIV envelope. While neither virus naturally infects nonhuman primates, the examination of viral distribution, penetration, and infection of viruses containing HIV envelope is crucial to understanding the translational relevance in humans. The explanation of using SHIV can be found in lines 215-217: "*The use of SHIV-1157ipd3N4, which was isolated from a pediatric HIV patient, allows for a translationally relevant model for investigating lentiviral pathogenesis of subclade C envelope, which is most dominant HIV subclade in sub-Saharan Africa [32]."

7. Discussed throughout the manuscript as well as at end of discussion (Line 438-440): From the paper "Furthermore, we also show that the entire GI tract is susceptible to viral infection after oral viral exposure. Our data indicate that the small intestine is the primary site for viral infection and that CD4+ T cells are the primary target cells 96 hours after viral exposure." The conclusion that the small intestine is the primary transmission point is difficult to conclude from the data provided. It seems since these findings provide some support, but some divergence, from previous studies (including Amadee et al 2018) the conclusions should reflect this. Also, data needs to be clearer and cleaner if the authors are to conclude that infection is occurring through the large intestines. As stated above, most of the data supporting this in the manuscript can also be explained as the virus or viral reporter simply being moved through the digestive tract with the formula. Suggest altering this conclusion.

Thank you for this suggestion. We have added discussion regarding this suggestion in lines 395-404: "Our findings are in congruence with the findings of previous studies, including Amedee et.al. who examined locations of SIV RNA and DNA over 96 hours, which showed that the ileum was the location with the second highest concentration of SIV RNA. However, in this study, it was reported that the location with the highest SIV RNA and DNA were the retropharyngeal lymph nodes [11]. It is important to note that this study examined SIV RNA and DNA only, which could possibly explain the differences observed in our study; likewise, we did not examine the retropharyngeal lymph nodes, which could be of interest in a future study. The observation that viral RNA could be detected in the iIleum and colon 96 hours after oral challenge is consistent with the abundant foci of infection detected in the gut 96 hours after challenge."

We have also softened our language in our conclusion to "Our data indicate that the small intestine has the potential to be a primary site for viral infection and that CD4+ T cells are the primary target cells 96 hours after viral exposure," *on lines 584-587 to reflect the concern expressed here.*

8. The title is not accurately describing the findings presented in the manuscript. One suggestion for a new

title might be: Detection of SHIV and viral reporters within the gastrointestinal tract following oral infection of neonatal rhesus macaques

Thank you for the suggestion for a new title. However, we believe that the current title accurately depicts what our study shows.

Part III – Minor Issues: Editorial and Data Presentation Modifications
Please use this section for editorial suggestions as well as relatively minor modifications of existing data that would enhance clarity.

Reviewer #1: 1. with such a small number of infants included in each necropsy study, etc - each data point on virion frequency, etc should be reported per animal, not averaged

Please see Part 2, Reviewer 1, Number 2 above: "HIV acquisition is inefficient and stochastic. For budgetary reasons the number of animals is small, but reveal where the virus CAN go, and remain intact, after an oral exposure. That is all we are trying to conclude from these initial feasibility studies. In the different analyses we used different mixed-effect models, as described in the methods, always using each animal as random effect to control for the lack of independence of the data sampled within each animal. Therefore, the results reflect and account for this fact. Afterwards, within the different models used and depending on the nature of the data analyzed, we performed all possible contrasts between every location within such fitted models to analyze statistically significant differences in the number of virions and/or their characteristics in each location. In order to do so, we used the Benjamini-Hochberg (BH) Procedure, as described in the methods, which controls for False Discovery Rate (FDR). On the contrary to what the reviewer questions about significant p-values for only 4 animals, when using BH, there is no reason why the sample size is relevant in expecting or not significant differences, as it is just a method to avoid Type I errors. Moreover, the power of our analysis is derived from the extensive sampling of virions in different tissues through multiple images from the animals, calculating averages per animals would defeat that purpose, gives us a very significant result without proper testing, and wouldn't allow us to test for our main interest, the differential anatomical distribution of the virions after mucosal exposure."

We have also added the total sum of virions found in each individual animal for the different anatomical groupings of the oral mucosa and GI tract. These results can be found throughout the text between lines 170-181.

2. authors should address the potential limitation with the dual infections of competition between the 2 viruses

Thank you for this statement. While there is the possibility of competition between the two viruses using this dual infection model, we found minimal evidence of LICh transduced cells in our tissues after oral challenge. This suggests that while we previously showed that LICh reporter virus was effective in transducing cells and identifying which cells are most susceptible to becoming the first cells infected in the female reproductive tract (Stieh et.al. 2014, Stieh et.al. 2016), and after rectal challenge (Maric D et al 2021). Although LICh is not as effective as a molecular beacon compared to the female reproductive tract. The effectiveness of LICh as a molecular beacon is even poorer after oral viral challenge. These differences are complex and a function of the efficiency of photons to pass through the tissue, the depth of the transduced cells, and the density of transduced cells, and background luminosity of alimentary canal tissues. Luckily it doesn't matter because we will utilize 64Cu labeled FAB2 probes and PET/CT targeted necropsy to identify sites of viral replication. As to the possibility of competition, our ability to visualize individual viral particles penetrating the tissue are at a density that would make virions compete. Viral particles appear to be limiting even under the high challenge dose condition of our studies. We did not mention this in the manuscript, but our initial efforts to utilize LICh alone in this neonate model was very

inefficient. No competition happening here.

3. in Discussion, include a reference for the paucity of CCR5+ CD4+ T cells in tonsils

Thank you for this comment. We have added two references (Doitsh et.al. and Finkel et.al.) on lines 430-440 to discuss the paucity of susceptible T cells in the tonsils. We need to educate the HIV research community about this key physiological aspect of HIV target cells in lymphatic tissues.

4. The handwriting on images is difficult to read - can the image include text boxes for indicating the anatomic locations?

Thank you for this comment. The handwriting on the images were notes from tissue processing after necropsy and tissue handling, not for presentation.

Reviewer #2: 1. In the Abstract, the first sentence is missing some words.

Thank you for this information. The first sentence has been edited.

2. Figure 1: Please explain what area of the picture the insert in Panel C refers to.

Thank you for this suggestion. The virion shown in the inset is now indicated with a white arrow, while other virions in the micrograph are indicated by grey arrows. This description has also been added to the Figure Legend.

3. Figure 2, Panel E: The mean values are barely readable.
Figure2, Panel F: Please clarify that the frequencies refer to total frequencies of virions counted at the specific anatomic site over the 2- and 4-hour period combined. The Panel could be deleted because the data represent the results of Panel E in a different format.

The point of this figure is to illustrate and quantify the difference in the distribution of the 64Cu signal at the 2- and 4-hour timepoints. Yes, the frequency is the to total frequencies of virions counted at the specific anatomic site over the 2- and 4-hour period combined. We have made that clearer in the figure legend.

4. Figure 3: The red background signal of Cell 1 in Panel A is not visible to the reviewer. Similarly, the colors in Panel C are difficult to distinguish. However, it is possible that the figures were not reproduced at print quality for the review stage.

Thank you for this comment. Figure 3 has been removed from the manuscript as per the suggestion from multiple reviewers.

5. Please check the reference section, most journal abbreviations are not listed correctly.

Thank you for this statement. We have followed the guidelines for Plos Pathogens references, using the Vancouver style in EndNote, which has the journal abbreviations preset.

6. It is probably not necessary to list the initials of the individuals who prepared the virus stock, but the decision lies with the authors.

Thank you for this suggestion. The initials of the authors have been removed from Supplemental Table 1 to

decrease distraction and irrelevant information from the table.

Reviewer #3: Minor comments:
• Line 179: Please replace “neck lymph nodes” with anatomically accurate definitions and replace (possibly cervical lymph nodes)

We have replaced all “neck lymph nodes” with “cervical lymph nodes” throughout the manuscript and in Table 1 to reflect correct anatomical definitions.

• Figure 1C is not labelled with a ‘C’

Thank you for this comment. This correction was made to Figure 1C.

• Line 423, and anywhere prior: “other T cells” should be more accurately referred to as CCR6- CD3+ T cells

Thank you for this comment. These changes have been made throughout the manuscript and the figures.

Sincerely yours,

Thomas J. Hope, PhD

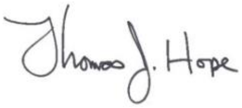

Professor, Northwestern University
Director, Viral Pathogenesis Core, Third Coast Center for AIDS Research
